# Leaf-chewing herbivores affect preference and performance of a specialist root herbivore

**DOI:** 10.1007/s00442-022-05132-9

**Published:** 2022-02-22

**Authors:** Peter N. Karssemeijer, Laura Winzen, Joop J. A. van Loon, Marcel Dicke

**Affiliations:** Laboratory of Entomology, Wageningen University and Research, Wageningen, The Netherlands

**Keywords:** Plant-mediated interactions, Root herbivory, *Delia radicum*, Plant defence, Oviposition, Preference–performance

## Abstract

Plants interact with a diversity of phytophagous insects above- and belowground. By inducing plant defence, one insect herbivore species can antagonize or facilitate other herbivore species feeding on the same plant, even when they are separated in space and time. Through systemic plant-mediated interactions, leaf-chewing herbivores may affect the preference and performance of root-feeding herbivores. We studied how six different leaf-chewing herbivore species of *Brassica oleracea* plants affected oviposition preference and larval performance of the root-feeding specialist *Delia radicum*. We expected that female *D. radicum* flies would oviposit where larval performance was highest, in accordance with the preference–performance hypothesis. We also assessed how the different leaf-chewing herbivore species affected defence-related gene expression in leaves and primary roots of *B. oleracea*, both before and after infestation with the root herbivore. Our results show that leaf-chewing herbivores can negatively affect the performance of root-feeding *D. radicum* larvae, although the effects were relatively weak. Surprisingly, we found that adult *D. radicum* females show a strong preference to oviposit on plants infested with a leaf-chewing herbivore. Defence-related genes in primary roots of *B. oleracea* plants were affected by the leaf-chewing herbivores, but these changes were largely overridden upon local induction by *D. radicum*. Infestation by leaf herbivores makes plants more attractive for oviposition by *D. radicum* females, while decreasing larval performance. Therefore, our findings challenge the preference–performance hypothesis in situations where other herbivore species are present.

**Supplementary Information:**

The online version contains supplementary material available at 10.1007/s00442-022-05132-9.

## Introduction

Plants are members of complex and diverse ecological communities, and in natural and agricultural settings alike, they are under attack by insect herbivores above- and belowground. In nature, pest outbreaks are rare, and herbivory may even safeguard biodiversity by decimating dominant plant species (Carson and Root 2000; Koerner et al. 2018). In agriculture, however, farmers suffer significant crop losses from insect herbivory (Deutsch et al. 2018; Oerke 2006). Understanding how plants defend themselves may provide new information for plant breeders to select crop varieties that are better able to resist herbivory.

Upon recognition of attack by an insect herbivore, induced defences are activated. These induced responses are regulated by a network of phytohormones, in which jasmonic acid (JA) is a central player (Erb and Reymond 2019; Pieterse et al. 2009). Cues that trigger plant defence can be general, such as mechanical wounding of a leaf or root, or more specific, such as the recognition of insect saliva at the wounding site (Acevedo et al. 2015). Through recognition, plants are able to fine-tune their response to a variety of phytophagous insects. There is great diversity in insect herbivores, and one distinguishing feature between them is diet breadth. Specialist insect herbivores feed on a single plant species or family, whereas generalists feed on plants from many phytochemically unrelated families. While specialists often evolved strategies to detoxify plant toxins or even use them for their own benefit, generalists rely on broad-spectrum detoxification enzymes or behavioural adaptations such as feeding on older leaves to cope with host plant defences (Abdalsamee and Müller 2012; Müller et al. 2001; Ratzka et al. 2002). Because generalist and specialist insect herbivore species differ in their strategies for overcoming host defence, it has been suggested that induction of plant defence may also be different, although evidence for this is limited (reviewed in Ali and Agrawal (2012)).

In natural settings, plants are often attacked by multiple species of insect herbivores (Stam et al. 2014). By defending against one herbivore species, defence against a second species may be altered. Hence, insect herbivores can interact with each other via induced plant defence, even when they are separated in time and space. Indeed, insect herbivory early in the season can affect the community of insects surrounding that plant even after the initial attacker is gone (Poelman et al. 2008). Factors such as feeding site and feeding mode of the inducing herbivore are important in determining whether facilitation or antagonism between herbivores occurs (Stam et al. 2014). Plant defence is not only triggered locally, but systemically throughout the plant. For instance, leaf herbivory in maize triggers defence signalling in roots of maize plants (Ankala et al. 2013). Since defence induction occurs systemically, plant-mediated interactions can also cross the shoot–root barrier. Indeed, leaf herbivores can affect the performance of root herbivores (Johnson et al. 2012; Kutyniok and Müller 2012).

A long-standing hypothesis in the field of entomology states that the oviposition preference of insects should be linked with the performance of their offspring: the so-called “mother knows best” or preference–performance hypothesis (Jaenike 1978; Johnson et al. 2006). To maximize fitness, female insects are expected to lay their eggs on the most suitable host plant for their larvae. A meta-analysis confirmed the hypothesis in situations without natural enemies or potential competitors (Gripenberg et al. 2010). However, most studies on the preference–performance hypothesis focus on insects with an aboveground life cycle, and whether this hypothesis also holds for root herbivores has received much less attention (Clark et al. 2011; Johnson et al. 2006; Menacer et al. 2021). Furthermore, oviposition preference may be altered by the presence of another insect, either directly or via induced plant defence. For example, *Pieris brassicae* butterflies prefer to lay eggs on uninfested leaves rather than leaves already infested with other caterpillars (Blaakmeer et al. 1994).

In this study, we test whether preference and performance of cabbage root fly *Delia radicum* are affected by leaf-chewing herbivores. Oviposition selection behaviour has been studied in great detail for *D. radicum*: a specialist root herbivore of brassicaceous plants (Schoonhoven et al. 2005). Brassicaceous plants produce glucosinolates (GSLs), which, upon attack, are hydrolysed by separately stored myrosinases to form breakdown products such as isothiocyanates or nitriles (Hopkins et al. 2009). Female *D. radicum* flies are attracted to cues from cabbage plants, such as volatile isothiocyanates derived from aliphatic GSLs (Hawkes and Coaker 1979). After landing on a leaf, the female inspects the chemical profile with taste receptors on her tarsi (Roessingh et al. 1992, 1997). If a host plant is accepted, the fly lays her eggs in the soil next to the plant stem (Schoonhoven et al. 2005). The cues used in this behaviour can be altered by prior herbivory, allowing female flies to integrate information about other herbivores to make oviposition choices.

In our experiments, we induced *Brassica oleracea* (Brussels sprouts) plants using six species of leaf-chewing herbivores, including both specialist and generalist species from several insect orders. For two of the species of leaf herbivores tested, *Plutella xylostella* and *P. brassicae*, plant-mediated antagonism towards root-feeding *D. radicum* larvae was previously described (Karssemeijer et al. 2020; Soler et al. 2007). To assess potential mechanisms underlying these interactions, we studied the expression of defence-related genes. Based on earlier experiments, we selected three genes that are strongly affected by *D. radicum* herbivory: *AOS* as a marker for JA biosynthesis, *CYP81F4* as a marker for indole GSL biosynthesis and *MYB28* as a marker for aliphatic GSL biosynthesis. Indole and aliphatic GSLs form different breakdown products upon hydrolysis and those formed from aliphatic GSLs are considered to be more toxic to chewing insect herbivores (Hopkins et al. 2009; Jeschke et al. 2015, 2016). We previously discovered that *D. radicum* larvae induce JA and indole GSL biosynthesis while suppressing aliphatic GSL biosynthesis (Karssemeijer et al., unpublished results). We assessed shoot dry mass to see whether leaf herbivore treatments had a major impact on plant health.

Based on previous studies (Karssemeijer et al. 2020; Soler et al. 2007), we hypothesize that leaf herbivores have a negative effect on the performance of *D. radicum*. We expect female *D. radicum* flies to lay more eggs on uninfested control plants than on plants infested with leaf herbivores, because we expect larval performance to be higher on uninfested plants. We expect that leaf herbivores differ in plant defence induction and plant-mediated effects on *D. radicum* preference and performance, and that these differences can be at least partly explained by the diet breadth of the inducing herbivore species.

## Materials and methods

### Study system

Three-week-old Brussels sprouts plants (*Brassica oleracea* L. var. *gemmifera* cv. Cyrus) were used in all experiments. Seeds were sown in trays and transplanted after seven days into 8 × 8 cm pots. Experiments were performed in a greenhouse compartment (L16:D8 photoperiod, 22 ± 2 °C, 50–70% relative humidity).

We used six species of leaf-chewing herbivores in the experiments. Two species of generalist herbivores, the cabbage moth [*Mamestra brassicae* L. (Lepidoptera: Noctuidae)] and silver Y [*Autographa gamma* L. (Lepidoptera: Noctuidae)], and two species of specialist herbivores, the large cabbage white [*Pieris brassicae* L. (Lepidoptera: Pieridae)] and diamondback moth [*Plutella xylostella* L. (Lepidoptera: Plutellidae)], were reared on Brussels sprouts plants. Two more species of specialist herbivores, the turnip sawfly [*Athalia rosae* L. (Hymenoptera: Tenthredinidae)] and mustard beetle *Phaedon cochleariae* Fabricius (Coleoptera: Chrysomelidae)], were reared on radish plants (*Raphanus sativus*). The species we defined as specialist feed on various brassicaceous plant species. The generalist species *M. brassicae* and *A. gamma* have been recorded to feed on at least 22 and 51 plant families, respectively (Maceljski and Balarin 1972; Rojas et al. 2000). Notably, *M. brassicae* is mostly a pest on brassicaceous plants. In the experiments, we used neonate larvae of *M. brassicae* and *P. brassicae*, and L1–L2 larvae of the other four species.

The cabbage root fly [*Delia radicum* L. (Diptera: Anthomyiidae)], a specialist herbivore, whose larvae feed on primary roots of brassicaceous plants, was reared on rutabaga (*B. napus* L. var. *napobrassica*). Flies were kept in cages (65 × 65 × 65 cm, Bugdorm, Taiwan), where they were provided with water, honey, and a 1:1:1 mixture of nutritional yeast, sugar, and milk powder. Eggs were collected by placing a slice of rutabaga in a Petri dish in the cage for several hours before taking it out and sealing it with Parafilm. After four days, neonate larvae hatched to be used for the experiments.

### Plant treatments

**Fig. 1:**
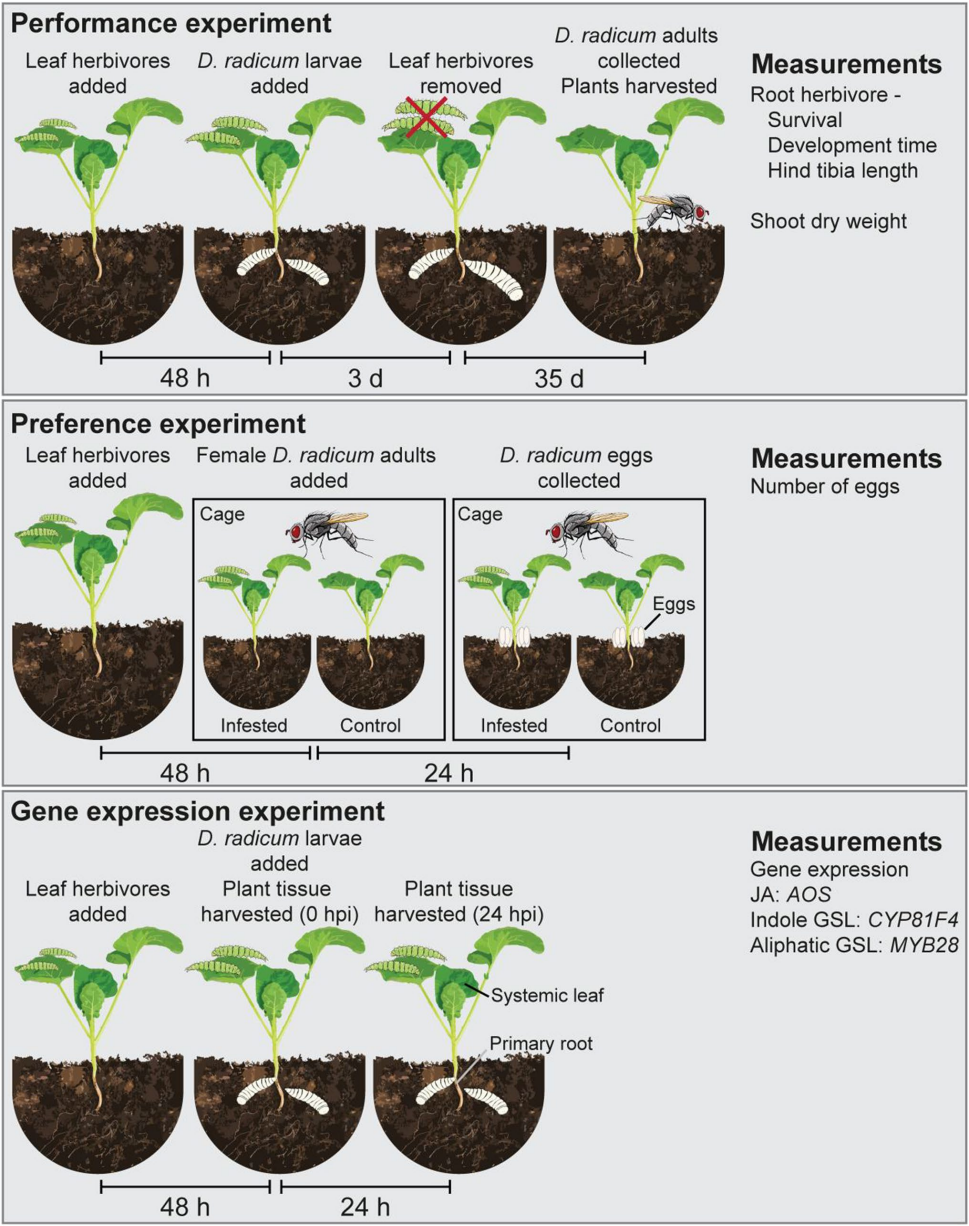
Experimental setup of the three experiments performed for this study to investigate the effects of leaf-chewing herbivores on the root herbivore *Delia radicum*. *GSL* glucosinolate, *JA* jasmonic acid, *hpi* hours post-infestation by *D. radicum*. Insect illustrations by Dr. Yidong Wang

**Fig. 2:**
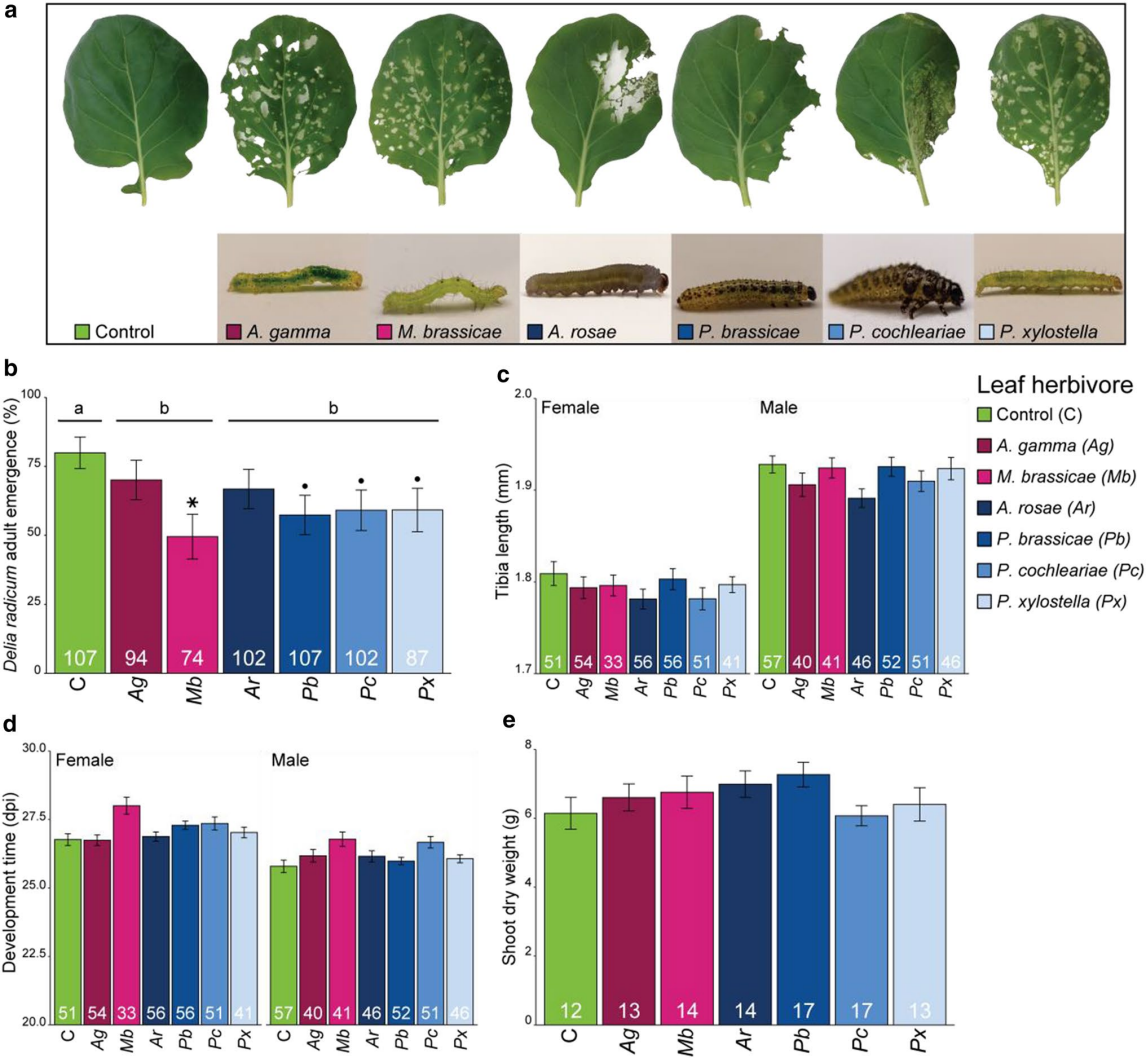
**a** Exemplary photographs of leaf damage by six species of leaf-chewing herbivores after 5 days of feeding on Brussels sprouts plants (*Brassica oleracea*). **b** *Delia radicum* adult emergence; **c** *Delia radicum* adult tibia length; **d** *Delia radicum* development time from egg until adult when feeding on plants previously subjected to leaf herbivory by one of six different leaf-chewing herbivore species. **e** Shoot dry weight at the end of the experiment. Leaf herbivores were placed on plants 2 days prior to *D. radicum* infestation and removed after they fed for 5 days. Magenta colours indicate generalist herbivores, whereas blue colours indicate specialist leaf herbivores of Brassicaceae. Stars (*P* < 0.05) and dots (*P* < 0.10) indicate differences between individual treatments and control, corrected for multiple testing using the false discovery rate method. Results of pairwise comparisons between herbivore specialization groups are indicated with letters; groups having no letters in common differ significantly (*P* < 0.05). Bars represent mean and error bars represent the standard error of the mean. Number of replicates are indicated in the bars. Leaf pictures by Laura Winzen, and larvae pictures by Peter Karssemeijer

We performed three experiments, in which plants were induced in a similar manner, to test effects of leaf-chewing herbivores on root herbivore performance, preference, and the induction of plant defence (Fig. 1). In each of the experiments, 3-week-old plants were treated by placing leaf-chewing herbivore larvae on the youngest fully expanded leaf. The petiole of this leaf was wrapped in cotton wool to prevent larvae from immediately moving to other leaves. We used ten larvae of *A. gamma*, *M. brassicae*, *P. cochleariae*, and *P. xylostella*, four *A. rosae* larvae or five *P. brassicae* larvae, to obtain roughly similar amounts of feeding damage (Fig. 2a). Control plants were not treated with any leaf herbivores, but did receive a piece of cotton wool around the petiole. During the experiments, plants were placed on saucers and watered from the bottom as needed, and provided 50 mL of Hyponex (Unifarm, Wageningen) fertilizer twice weekly.

### *Delia radicum* performance

To test the effect of feeding by different leaf herbivore species on root herbivore performance, 24 plants per treatment were prepared as explained above. In addition to the cotton wool, small mesh bags were secured around the induced leaf to prevent larvae from escaping. As this experiment lasted more than a month, the soil was covered with a layer of sand to reduce attractiveness to fungus gnats (Diptera: Sciaridae). Two days after leaf infestation, ten neonate *D. radicum* larvae were placed directly on the hypocotyl of all plants, including control plants. Three days post-infestation (dpi) with *D. radicum* larvae, the mesh bags and leaf herbivores were removed. At 20 dpi, mesh bags were placed around each plant and sealed with a rubber band around the top of the pot. Bags were held up by two wooden sticks in the soil to give plants space to grow.

The first flies were observed at 24 dpi, but some may have emerged 1 or 2 days earlier in the weekend. After the first flies had emerged, every plant was checked daily for emergence. Flies were collected using an aspirator and stored in a freezer (− 18 °C) after recording their sex and day of emergence. The experiment was terminated at 38 dpi. As an estimation of body size, the hind tibia length was measured using a digital microscope (Dino Lite Edge, Taiwan) (Soler et al. 2007). After the experiment, we harvested the aboveground plant parts, dried them in an oven for 7 days at 70 °C, and assessed the shoot dry weight (Sartorius CPC2 balance, Germany).

During this experiment, an infestation with thrips occurred in the greenhouse. To minimize the effects of thrips on our experiment, we excluded badly thrips-damaged plants from the analyses. Further, in some cases, plants were damaged by chewers that were missed during removal, these plants were also discarded, resulting in 13–17 plants per treatment.

### *Delia radicum* preference

To study whether *D. radicum* oviposition preference is affected by leaf herbivory, we performed a two-choice experiment. Due to greenhouse space limitations, this experiment was performed in three rounds in subsequent weeks. For *A. gamma* and *M. brassicae*, data were collected in two rounds due to difficulties in synchronizing the rearing. In each round, we repeated each treatment five to six times, resulting in 11–16 replicates per treatment in total. Six weeks prior to each round, a separate *D. radicum* rearing cage was started. In short, eggs were collected as above and placed on halved rutabagas at a density of one egg per gram of root (Dr. Anne-Marie Cortesero, pers. Com.), which were placed on a layer of sand in a plastic tray. Four weeks later, pupae were collected from the sand by sieving and placed in cages (65 × 65 × 65 cm, Bugdorm, Taiwan) in a separate climate cabinet to minimize rutabaga scent exposure of emerging flies. Female flies used in the experiment were 1–2 weeks old, giving them enough time since pupation to mate and develop eggs.

Three-week-old Brussels sprouts plants were treated as above. As an oviposition substrate from which the eggs could later be extracted, we covered the top layer of soil with roughly 3 cm of white sand. Two days after leaf treatment, we prepared two-choice arenas in foldable cages (60 × 40 × 40 cm). In each tent, an untreated control plant was placed on one side, and an infested plant on the other, roughly 40 cm apart. Control cages had only untreated plants. In each cage, food (1:1:1 mixture nutritional yeast, sugar, and milk powder) and water were provided. We randomized positions of cages in the greenhouse compartment and positions of control and infested plants within each cage. To start the oviposition trial, five gravid *D. radicum* female flies were collected using an aspirator in a 50 mL tube, which was placed in the centre of each cage and opened. After 24 h, plants were removed from the cages, and the layer of white sand with the eggs was collected in plastic boxes by holding the pot sideways and gently tapping. Eggs stuck to the stems of plants were collected using a brush. Boxes were stored at 7 °C until further processing. Eggs were separated from sand by flotation using a Fenwick can and a 500 µm sieve and immediately counted (Fenwick 1940).

### Gene expression

We tested how leaf and root herbivores affected transcript levels of genes related to plant defence. To this end, we treated plants as above with six species of leaf herbivores that were prevented from moving to other leaves by securing small mesh bags around the petioles. Plants were divided into two subsets. The first subset was harvested 2 days after induction by leaf herbivores; this time point corresponds with the time of infestation by *D. radicum* in the performance experiment (0 hpi) and the time that plants were presented to female *D. radicum* flies for the preference experiment (Fig. 1). The second subset of plants was infested with ten *D. radicum* larvae after 2 days of leaf feeding and harvested 24 h later. At this time point, preliminary experiments had shown strong induction of the tested genes by the root herbivore. An additional control treatment that did not receive root herbivores was included in this subset.

At the time of harvesting, plants were uprooted and the primary root was cut off using clean scissors. Samples were immediately wrapped in aluminium foil and frozen in liquid nitrogen. At the same time, systemic leaf tissue (one leaf higher than the infested leaf) was collected from one leaf higher than the induced leaf using a 10 mm cork borer. Three leaf disks were harvested from the same leaf, placed in an Eppendorf tube, and frozen in liquid nitrogen. Tissue from three plants was pooled for each replicate, and four replicates were taken per treatment and time point. Harvesting took less than 1 min per sample.

Root and shoot tissue was ground using mortar and pestle or plastic Eppendorf pestles, respectively, whilst keeping the sample cold in liquid nitrogen. From this ground tissue, RNA was extracted using the Isolate II Plant RNA kit (Bioline) and converted to cDNA using the SensiFAST cDNA synthesis kit (Bioline). We then performed qPCR using SensiFAST SYBR (Bioline), targeting defence-related transcripts *AOS*, *CYP81F4*, and *MYB28* (Table S1). We selected *AOS* as a marker for JA biosynthesis, *CYP81F4* as a marker for indole GSL biosynthesis, and *MYB28* as a marker for aliphatic GSL biosynthesis. The latter two genes were previously found to be among the most strongly affected genes in the primary root response to *D. radicum* (Karssemeijer et al., unpublished results). Optimal reference genes were selected based on GeNorm analysis, in which a random subset of 16 root or leaf samples were tested for six reference genes (Vandesompele et al. 2002). For root tissue, *Act-2* and *PER4* were used as reference genes, and for leaf tissue *Act-2* and *SAR1a* were used. Three mixes of samples were included on each qPCR plate as interrun calibrators. Relative expression was calculated in qBase + (Biogazelle, Belgium), taking into account primer efficiency and interrun calibration.

### Statistics

Data was analysed using R version 3.6.3 (R Core Development Team 2017) with packages lme4 (Bates et al. 2015), emmeans (Lenth et al. 2018), lmtest (Zeileis and Hothorn 2002), RVAideMemoire (Hervé 2020), and multcomp (Hothorn et al. 2008). Depending on the type of data (i.e. counts or continuous values) and normality, (generalized) linear (mixed) models ((G)L(M)Ms) were used for data analysis with normal, gamma, poisson, negative binomial or binomial distributions. We selected the best model that included all factors of interest based on Akaike information criteria (AIC) values.

In the analysis of *D. radicum* emergence (GLMM with binomial distribution), tibia length (LMM) and development time (GLMM with Poisson distribution), each fly was considered a replicate and plant ID was included as a random factor to avoid pseudoreplication. For oviposition choice (GLMM with binomial distribution), experimental round and cage ID were included as random factors. To determine whether treatments significantly affected *D. radicum* oviposition choice, we tested whether the fraction of eggs laid on control versus infested plants differed between cages with and without an infested plant. For the sake of this analysis, one of the two uninfested control plants in each control cage received the arbitrary label “infested”. For the number of eggs per cage (GLMM with negative binomial distribution), experimental round was included as a random factor. Relative gene expression was analysed using GLM with a gamma distribution or LM depending on normality.

For each model, we performed pairwise comparisons with two methods. First, we compared each leaf herbivore treatment to the control treatment. Secondly, we ran a pairwise comparison between control, generalist leaf herbivores and specialist leaf herbivores using the user.cont() function.

## Results

### Leaf herbivory affects *D. radicum* emergence

We assessed *D. radicum* adult emergence as a proxy for survival on plants induced by different leaf-chewing herbivores (Fig. 2b). While the effect of individual treatments on the emergence of *D. radicum* was weak (GLMM; *χ*^2^ = 10.54, *P* = 0.10), comparison of the different herbivore treatments to the control treatment revealed that feeding by *M. brassicae* reduced *D. radicum* emergence, and there was a trend that *P. brassicae* (*Z* = 2.36, *P* = 0.055), *P. cochleariae* (*Z* = 2.17, *P* = 0.055), and *P. xylostella* (*Z* = 2.09, *P* = 0.055) slightly reduced root fly emergence. Pairwise comparisons of herbivores grouped by their dietary breadth showed that both generalist and specialist leaf-chewing herbivores negatively affected *D. radicum* emergence compared to the control treatment. Hind tibia length of emerged flies was not affected by leaf herbivore treatments (Fig. 2c, LMM; *χ*^2^ = 14.18, *P* = 0.028). Males had longer hind tibia than females (LMM; *χ*^2^ = 315.58, *P* < 0.001). Development time of cabbage root flies was not affected by leaf herbivores (Fig. 2d, GLMM; *χ*^2^ = 2.57, *P* = 0.86). Males emerged earlier than females (GLMM; *χ*^2^ = 5.04, *P* = 0.025). Shoot dry weight of the plants on which flies developed was not affected by leaf herbivory (Fig. 2e, LMM; *χ*^2^ = 7.97, *P* = 0.24).

### Female *D. radicum* flies prefer to lay eggs on plants damaged by leaf herbivores

**Fig. 3:**
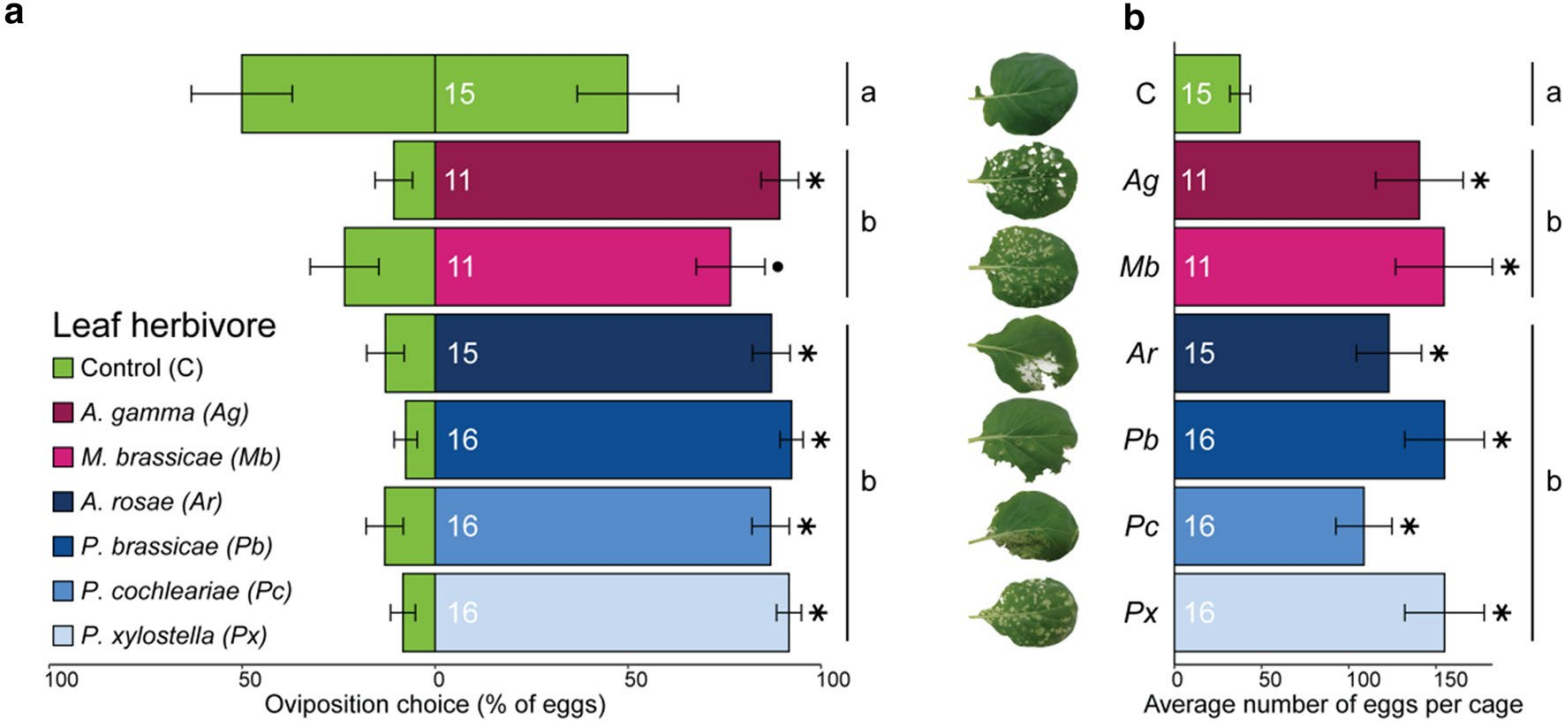
**a** *Delia radicum* oviposition choice when choosing between a plant previously subjected to leaf herbivory by six different leaf-chewing herbivore species or an uninfested control plant and **b** the average total number of eggs per cage. Leaf herbivores were placed on plants 2 days prior to the start of the two-choice assay. Five *D. radicum* females were released in each cage and taken out 24 h later. Magenta colours indicate generalist herbivores, whereas blue colours indicate specialist leaf herbivores of Brassicaceae. Stars (*P* < 0.05) and dots (*P* < 0.10) indicate differences between individual treatments and control, corrected for multiple testing using the false discovery rate method. Results of pairwise comparisons between herbivore specialization groups are indicated with letters; groups having no letters in common differ significantly (*P* < 0.05). Bars represent mean and error bars represent the standard error of the mean. Number of replicates are indicated in the bars

We studied the effects of leaf herbivory on oviposition preference of *D. radicum* using two-choice assays. Female *D. radicum* flies strongly preferred to lay eggs at the base of plants infested by leaf herbivores, both specialists and generalists (Fig. 3a, GLMM; *χ*^2^ = 19.09, *P* = 0.0040). There was an effect of leaf herbivore treatments on *D. radicum* oviposition, and indeed, except for *M. brassicae*, feeding by each of the leaf herbivore species led to more *D. radicum* eggs compared to the control. The effect was less strong for *M. brassicae* (*Z* = 1.77, *P* = 0.077), which may be due to a smaller sample size for this treatment. We also analysed the sum of eggs per cage (control and induced plants combined) and found that many more eggs were laid in cages that contained a plant infested with leaf herbivores compared to control cages (Fig. 3b, GLMM; *χ*^2^ = 43.52, *P* < 0.001).

### Effects of leaf herbivores on the expression of plant defence genes in systemic leaves and in roots in the absence of *D. radicum*

**Fig. 4:**
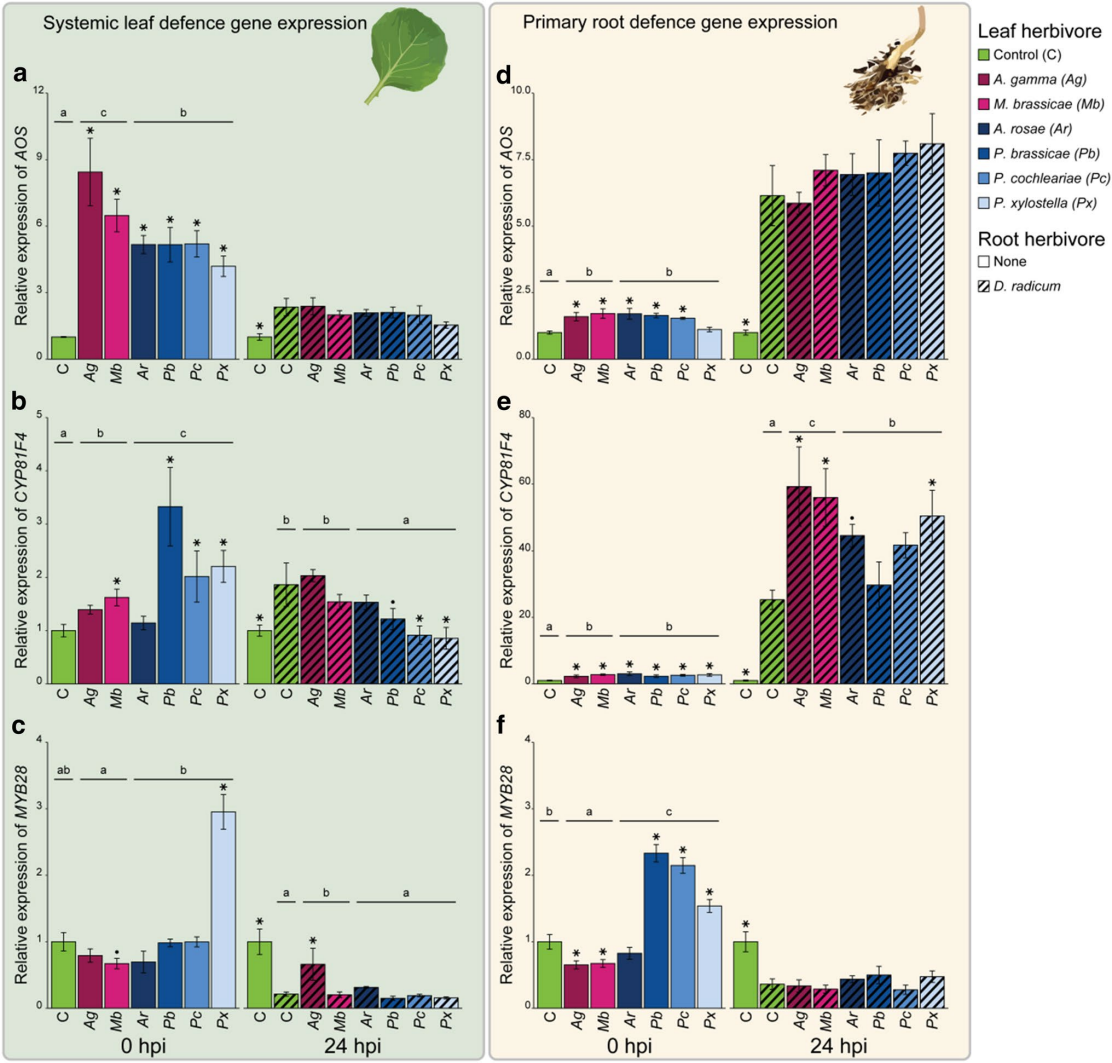
Defence gene expression in systemic leaves (left panel, **a**–**c**) and primary roots (right panel, **d–f**) of *Brassica oleracea* plants infested with six species of leaf-chewing herbivores either alone or in combination with the root herbivore *Delia radicum*. Systemic leaves were one leaf higher than the leaf infested with leaf herbivores. Leaf herbivores were placed on plants 2 days prior to *D. radicum* infestation. Magenta colours indicate generalist herbivores, whereas blue colours indicate specialists of the Brassicaceae family. Stars (*P* < 0.05) and dots (*P* < 0.1) indicate differences between individual treatments and control plants (0 hpi) or plants only infested with *D. radicum* (24 hpi), corrected for multiple testing using the false discovery rate method. Note that symbols above uninfested control plants at 24 hpi indicate a difference compared to *D. radicum*-infested plants. Results of pairwise comparisons between herbivore specialization groups are indicated with letters; groups having no letters in common differ significantly (*P* < 0.05). Hpi: hours post-infestation by *D. radicum*. Bars represent mean and error bars represent the standard error of the mean. *N* = 4 replicates each consisting of three plants

In systemic leaves, transcript levels of *AOS, CYP81F4* and *MYB28* were affected by leaf herbivory at 0 hpi (Fig. 4a–c, *AOS*: GLM; *χ*^2^ = 59.53, *P* < 0.001, *CYP81F4*: GLM; *χ*^2^ = 32.65, *P* < 0.001, *MYB28*: GLM; *χ*^2^ = 53.56, *P* < 0.001). All species of leaf herbivores induced expression of *AOS*, leading to a 4- to 8.5-fold increase. Generalists had a stronger effect on *AOS* transcript levels than specialists. On the other hand, specialist herbivores caused stronger induction of *CYP81F4* compared to generalist herbivores. Expression of *CYP81F4* was induced by *M. brassicae*, *P. brassicae*, *P. cochleariae*, and *P. xylostella*, but not by *A. gamma* and *A. rosae*. Transcript levels of *MYB28* were higher in systemic leaves of plants induced by specialists compared to generalists. When analysing at the species level, this effect is mainly caused by a strong induction of the gene by *P. xylostella* and a slight downregulation by *M. brassicae* compared to uninfested control plants.

In primary roots at 0 hpi, expression of all three genes (*AOS, CYP81F4* and *MYB28*) was affected by leaf herbivory (Fig. 4d–f, *AOS*: LM; *χ*^2^ = 24.96, *P* < 0.001, *CYP81F4*: LM; *χ*^2^ = 18.74, *P* = 0.0046, *MYB28*: GLM; *χ*^2^ = 71.40, *P* < 0.001). Both generalist and specialist herbivores upregulated the expression of *AOS* as well as *CYP81F4*. Except for *P. xylostella*, each of the leaf herbivore species upregulated *AOS* expression relative to control. *CYP81F4* mRNA levels were induced in the roots by all six leaf herbivore species. Expression of *MYB28* in the primary roots at 0 hpi was induced by specialists but suppressed by generalist herbivores, compared to control roots. At the species level, the generalist chewers *A. gamma* and *M. brassicae* downregulated *MYB28* expression, whereas specialists *P. brassicae*, *P. cochleariae* and *P. xylostella* caused upregulation of this gene.

### Effects of leaf herbivores on plant defence genes after infestation with *D. radicum*

The three measured defence-related genes were affected by herbvore treatments in systemic leaves at 24 hpi (Fig. 4a–c, *AOS*: LM; *χ*^2^ = 18.27, *P* = 0.011, *CYP81F4*: LM; *χ*^2^ = 27.29, *P* < 0.001, *MYB28*: GLM; *χ*^2^ = 56.42, *P* < 0.001). Expression of *AOS* was induced by *D. radicum* infestation of the primary roots, and this effect was not altered when plants were infested with *D. radicum* in combination with generalist or specialist leaf herbivores. Expression of *CYP81F4* was induced by root herbivory. Specialist herbivores in combination with *D. radicum* led to a lower expression of *CYP81F4* compared to plants infested only with *D. radicum*. Further, we measured lower *CYP81F4* expression levels in systemic leaves induced by *P. cochleariae* and *P. xylostella* in combination with *D. radicum* compared to plants infested with *D. radicum* alone. *Delia radicum* reduced the expression of *MYB28* in systemic leaves. Plants co-infested with *M. brassicae* and *D. radicum* had higher expression levels of *MYB28* compared to systemic leaves of plants infested with *D. radicum* alone.

In primary roots, 24 h after *D. radicum* infestation, a clear effect of herbivore treatment was measured for all three genes (Fig. 4d–f, *AOS*: GLM; *χ*^2^ = 60.33, *P* < 0.001, *CYP81F4*: LM; *χ*^2^ = 39.12, *P* < 0.001, *MYB28*: GLM; *χ*^2^ = 25.40, *P* < 0.001). Primary root expression of *AOS* at 24 hpi was induced sixfold by *D. radicum* compared to control, but did not differ between plants infested with *D. radicum* alone or in combination with leaf herbivores. Expression of *CYP81F4* was strongly induced by 24 h of feeding by *D. radicum*. Furthermore, both specialist and generalist leaf herbivores had a synergistic effect with root herbivore induction, leading to higher expression of *CYP81F4* when compared to plants only infested with *D. radicum*. On the species level, higher levels of *CYP81F4* were measured in primary roots of plants infested with both *D. radicum* and *A. gamma*, *M. brassicae* or *P. xylostella* compared to plants only infested with the root herbivore. Primary roots that were infested with *D. radicum* for 24 h contained lower levels of *MYB28* mRNA compared to uninfested control. Expression of *MYB28* was not different between plants infested with *D. radicum* alone or in combination with leaf herbivores.

## Discussion

We studied plant-mediated effects of leaf herbivores on preference and performance of the root herbivore *D. radicum* in the context of two hypotheses in the field of insect–plant interactions, i.e. that oviposition preference is linked to higher larval performance and that generalists and specialists induce distinct plant responses. Both generalist and specialist leaf-chewing herbivores negatively affected *D. radicum*, in line with earlier findings that leaf chewers generally negatively affect root chewers (Johnson et al. 2012). We discovered that female *D. radicum* flies strongly preferred to lay eggs on plants exposed to leaf herbivory, even though larval performance was reduced on those plants.

### Leaf-chewing herbivores affect systemic plant defence and *Delia radicum* performance

Our results show that chewing folivores can affect *D. radicum* performance. While both specialist and generalist herbivores negatively affected *D. radicum* emergence when analysed as a group, these effects were only marginally statistically significant when analysing the effects of the individual species of inducing herbivores, showing that effects were relatively weak. Previously, negative plant-mediated effects of *P. brassicae* and *P. xylostella* on cabbage root fly emergence have been reported (Karssemeijer et al. 2020; Soler et al. 2007). Shoot dry weight was not affected by the leaf herbivores, suggesting that lower performance was not caused by reduced plant growth but rather by differences in induced plant defence. We found that leaf herbivores induced JA biosynthesis marker gene *AOS* in both systemic leaves and primary roots. Treatment with JA can be sufficient to reduce *D. radicum* performance, although contrasting results have been reported (Pierre et al. 2012). Induction of JA biosynthesis was also found in the primary roots of plants induced by *A. gamma*, while performance was not affected by this treatment. In primary roots, leaf herbivores caused an increase in expression of indole GSL biosynthesis gene *CYP81F4*, which was strengthened further 24 h after *D. radicum* started feeding. Transcription related to aliphatic GSL biosynthesis was found to be both up- and downregulated depending on the species of leaf-chewing inducer prior to onset of root herbivory. Aliphatic GSLs are suggested to be more effective against insect herbivores than indole GSLs due to differences in hydrolysis products (Jeschke et al. 2016). *Delia radicum* performs better on transgenic cabbage plants in which aliphatic GSL biosynthesis has been knocked down compared to wild-type plants (Karssemeijer et al., unpublished results), making this a potential mechanism for slightly reduced performance on plants induced by *P. brassicae*, *P. cochleariae*, and *P. xylostella*, in which *MYB28* expression was upregulated. However, after *D. radicum* started feeding, this upregulation was reversed, and all plants showed a decrease in *MYB28* transcription similar to roots only treated with *D. radicum*. Root herbivore-induced expression of indole GSL biosynthesis gene *CYP81F4* was synergistically increased by several leaf herbivores. The toxicity of indole GSLs to *D. radicum* larvae has not been studied, however, most likely it is low. In addition to the three genes we measured, other plant defence mechanisms may have been altered by leaf herbivores, such as myrosinase activity, mechanical resistance of the roots, or other defensive chemical compounds.

Our results show no clear evidence of distinct differences between generalist and specialist leaf herbivores in the induction of systemic plant defence or plant-mediated interaction with *D. radicum*. In terms of *D. radicum* preference and performance, effects of leaf herbivores were unidirectional, and differences appeared between individual species. For instance, *M. brassicae* and *A. gamma*, the two generalist species we used and also the closest genetic relatives in our study, caused the strongest and weakest effect on *D. radicum* emergence, respectively. Similar species-specific rather than specialization-driven plant-mediated interactions were reported between four species of leaf herbivores in wild radish (Agrawal 2000).

In terms of plant defence induction, generalist and specialist herbivores may induce distinct defence responses (Ali and Agrawal 2012; Rowen and Kaplan 2016). In systemic leaf gene expression, we indeed observe stronger induction of *AOS* by generalist herbivores compared to specialists, and the reverse for *CYP81F4*. On the other hand, our gene expression analyses point much more towards species-specific responses; for instance, in systemic leaves, *MYB28* expression was strongly induced only by *P. xylostella*, and *CYP81F4* expression was induced by all herbivore species except for *A. rosae* and *A. gamma*. This corresponds with earlier studies that failed to find distinct patterns of induction by generalists and specialists (Ali and Agrawal 2012; Bidart-Bouzat and Kliebenstein 2011; Reymond et al. 2004). For instance, microarray analysis of the *Arabidopsis thaliana* response to four chewing herbivores, two generalists and two specialists, revealed no effect of specialization, whereas the specialists *P. rapae* and *P. xylostella* elicited a very different response (Bidart-Bouzat and Kliebenstein 2011). Upon feeding, chewing herbivores release saliva and regurgitant that may include effector proteins to interact with plant defence (Acevedo et al. 2015), which could potentially explain species-specific responses. Further, some differences in effects of generalist versus specialist herbivores may be caused by the leaf age preferred for feeding. For instance, the specialist *P. xylostella* prefers to feed on the youngest developing leaves, whereas the generalist *M. brassicae* is found more often on older leaves. We excluded this effect from our study by constraining each herbivore to a single leaf, which may have masked differences between generalists and specialists.

### Leaf herbivory affects oviposition of *Delia radicum*

Female *D. radicum* flies strongly preferred to lay eggs on plants induced by leaf herbivores. The oviposition behaviour of *D. radicum* has been recorded in much detail (Schoonhoven et al. 2005; Zohren 1968). *Delia radicum* aggregates in the field (Mukerji and Harcourt 1970), and oviposition is stimulated on plants damaged by conspecifics, even when stems were cut and shoots were removed prior to testing (Baur et al. 1996). Plants infested with *Brevicoryne brassicae* or *Myzus persicae* aphids received fewer eggs by *D. radicum* females, possibly due to physical contact between searching flies and aphids (Finch and Jones 1989), as the opposite effect was observed when *B. brassicae* aphids were removed prior to the test (Finch and Jones 1987), although induction of plant defence by the aphids may also play a role. *Delia radicum* females were deterred from plants with *P. xylostella* eggs, but attracted to plants on which second or third instar larvae were feeding, in line with our findings (Finch and Jones 1987). Finally, spraying plants with extracts of frass of *Evergestis forficalis* caterpillars works as an oviposition deterrent for cabbage root flies (Jones et al. 1988). Specialist insect herbivores of Brassicaceae often use GSLs as oviposition stimulants (Textor and Gershenzon 2009). Indeed, indole as well as aliphatic GSLs are oviposition stimulants for *D. radicum* upon contact (Roessingh et al. 1992), and isothiocyanates that are produced upon aliphathic GSL hydrolysis are volatile attractants for gravid females (Hawkes and Coaker 1979). We found upregulation of GSL biosynthesis gene expression upon leaf herbivory by most species, and it is expected that constitutive GSLs stored in leaves are converted to isothiocyanates upon leaf damage (Textor and Gershenzon 2009). However, GSLs do not trigger the entire response; several compounds classified as cabbage identification factors (CIFs) induce a much stronger oviposition response than GSLs (de Jong et al. 2000; Roessingh et al. 1997). To the best of our knowledge, whether concentrations of CIFs differ upon herbivory has not been studied yet. In natural settings, there are many ways a plant can be mechanically damaged, such as by wind, heavy rain, or a rodent brushing past. Therefore, finding a completely undamaged plant like the control plants in our experiments is unrealistic in natural settings. Female *D. radicum* may not be adapted to recognize such plants as potential hosts, especially when stronger-smelling damaged plants are nearby.

Our finding that plants damaged by chewing folivores are more attractive for oviposition by *D. radicum* despite lower larval performance challenges the preference–performance hypothesis when plant-mediated interactions are considered (Jaenike 1978; Johnson et al. 2006). While many studies have supported this hypothesis (Gripenberg et al. 2010), there are exceptions. Menacer et al. (2021) recently found support for the preference–performance hypothesis in *D. radicum* when comparing between cultivars of *B. rapa*, but not between cultivars of *S. alba*. Furthermore, *Otiorhynchus sulcatus* vine weevils laid more eggs on raspberry plants previously damaged by conspecific larvae and on raspberry plants with lower root mass, even though both these factors negatively impacted larval mass (Clark et al. 2011). Our experiment investigated plant–herbivore interactions without the inclusion of natural enemies. *Trybliographa rapae* Westwood parasitoid wasps foraging for *D. radicum* larvae use volatile cues to locate hosts (Neveu et al. 2002). The presence of other herbivores may change the volatile blend, thereby reducing parasitism (Pierre et al. 2011; Rasmann and Turlings 2007). Indeed, leaf herbivory by *P. brassicae* leads to lower parasitism of *D. radicum* in the roots in both laboratory and field conditions (Pierre et al. 2011). Through lower parasitism, choosing for leaf-induced plants may yet be a beneficial strategy for *D. radicum* survival. To complement our greenhouse studies, preference and performance of *D. radicum* in the context of leaf herbivory should be studied in the field before firm conclusions can be made.

## Conclusion

Plants in natural or agricultural settings are often attacked by both above- and belowground insect herbivores, linking the two communities. Our results show that leaf herbivores strongly affect the oviposition preference of *D. radicum* females, while slightly decreasing larval performance. Leaf herbivores induced defence-related gene expression in both systemic leaf and root tissue, which was largely overridden after the induction of the root herbivore. Our findings highlight that through plant-mediated interactions, leaf herbivores can affect the oviposition choice of root herbivores, with potential consequences for insect community dynamics in the field. This presents a novel link between above- and belowground insect herbivore communities through changes in oviposition behaviour.

## Supplementary Information

Below is the link to the electronic supplementary material.

Supplementary file1 (DOCX 14 KB)

## Data Availability

The datasets used during the current study are available from the corresponding author on reasonable request.

## References

[CR1] Abdalsamee MK, Müller C (2012). Effects of indole glucosinolates on performance and sequestration by the sawfly *Athalia rosae* and consequences of feeding on the plant defense system. J Chem Ecol.

[CR2] Acevedo FE, Rivera-Vega LJ, Chung SH, Ray S, Felton GW (2015). Cues from chewing insects—the intersection of DAMPs, HAMPs, MAMPs and effectors. Curr Opin Plant Biol.

[CR3] Agrawal AA (2000). Specificity of induced resistance in wild radish: causes and consequences for two specialist and two generalist caterpillars. Oikos.

[CR4] Ali JG, Agrawal AA (2012). Specialist versus generalist insect herbivores and plant defense. Trends Plant Sci.

[CR5] Ankala A, Kelley RY, Rowe DE, Williams WP, Luthe DS (2013). Foliar herbivory triggers local and long distance defense responses in maize. Plant Sci.

[CR6] Bates D, Maechler M, Bolker B, Walker S (2015). Fitting linear mixed-effects models using lme4. J Stat Softw.

[CR7] Baur R, Kosal V, Patrian B, Stadler E (1996). Preference for plants damaged by conspecific larvae in ovipositing cabbage root flies: influence of stimuli from leaf surface and roots. Entomol Exp Appl.

[CR8] Bidart-Bouzat MG, Kliebenstein D (2011). An ecological genomic approach challenging the paradigm of differential plant responses to specialist versus generalist insect herbivores. Oecologia.

[CR9] Blaakmeer A, Hagenbeek D, van Beek TA, de Groot A, Schoonhoven LM, van Loon JJA (1994). Plant response to eggs vs. host marking pheromone as factors inhibiting oviposition by *Pieris brassicae*. J Chem Ecol.

[CR10] Carson WP, Root RB (2000). Herbivory and plant species coexistence: community regulation by an outbreaking phytophagous insect. Ecol Monogr.

[CR11] Clark KE, Hartley SE, Johnson SN (2011). Does mother know best? The preference–performance hypothesis and parent–offspring conflict in aboveground–belowground herbivore life cycles. Ecol Entomol.

[CR12] de Jong R, Maher N, Patrian B, Städler E, Winkler T (2000). Rutabaga roots, a rich source of oviposition stimulants for the cabbage root fly. Chemoecology.

[CR13] Deutsch CA, Tewksbury JJ, Tigchelaar M, Battisti DS, Merrill SC, Huey RB, Naylor RL (2018). Increase in crop losses to insect pests in a warming climate. Science.

[CR14] Erb M, Reymond P (2019). Molecular interactions between plants and insect herbivores. Annu Rev Plant Biol.

[CR15] Fenwick DW (1940). Methods for the recovery and counting of cysts of *Heterodera schachtii* from soil. J Helminthol.

[CR16] Finch S, Jones H, Labeyrie V, Fabres G, Lachaise D (1987). Interspecific competition during host plant selection by insect pests of cruciferous crops. Insect–plants, Proceedings 6th international symposium on insect-plant relationships.

[CR17] Finch S, Jones TH (1989). An analysis of the deterrent effect of aphids on cabbage root fly (*Delia radicum*) egg-laying. Ecol Entomol.

[CR18] Gripenberg S, Mayhew PJ, Parnell M, Roslin T (2010). A meta-analysis of preference–performance relationships in phytophagous insects. Ecol Lett.

[CR19] Hawkes C, Coaker TH (1979). Factors affecting the behavioural responses of the adult cabbage root fly, *Delia brassicae*, to host plant odour. Entomol Exp Appl.

[CR20] Hervé M (2020) R Package ‘RVAideMemoire’: see CRAN.R-project.org/package=RVAideMemoire

[CR21] Hopkins RJ, van Dam NM, van Loon JJA (2009). Role of glucosinolates in insect–plant relationships and multitrophic interactions. Annu Rev Entomol.

[CR22] Hothorn T, Bretz F, Westfall P (2008). Simultaneous inference in general parametric models. Biometrical Journal: Journal of Mathematical Methods in Biosciences.

[CR23] Jaenike J (1978). On optimal oviposition behavior in phytophagous insects. Theor Popul Biol.

[CR24] Jeschke V, Gershenzon J, Vassão DG, Jetter R (2015). Metabolism of glucosinolates and their hydrolysis products in insect herbivores. The formation, structure and activity of phytochemicals.

[CR25] Jeschke V, Gershenzon J, Vassão DG, Kopriva S (2016). Insect detoxification of glucosinolates and their hydrolysis products. Glucosinolates.

[CR26] Johnson SN, Birch ANE, Gregory PJ, Murray PJ (2006). The ‘mother knows best’ principle: should soil insects be included in the preference–performance debate?. Ecol Entomol.

[CR27] Johnson SN, Clark KE, Hartley SE, Jones TH, McKenzie SW, Koricheva J (2012). Aboveground–belowground herbivore interactions: a meta-analysis. Ecology.

[CR28] Jones TH, Cole RA, Finch S (1988). A cabbage root fly oviposition deterrent in the frass of garden pebble moth caterpillars. Entomol Exp Appl.

[CR29] Karssemeijer PN, Reichelt M, Gershenzon J, van Loon JJA, Dicke M (2020). Foliar herbivory by caterpillars and aphids differentially affects phytohormonal signalling in roots and plant defence to a root herbivore. Plant Cell Environ.

[CR30] Koerner SE (2018). Change in dominance determines herbivore effects on plant biodiversity. Nat Ecol Evol.

[CR31] Kutyniok M, Müller C (2012). Crosstalk between above- and belowground herbivores is mediated by minute metabolic responses of the host *Arabidopsis thaliana*. J Exp Bot.

[CR32] Lenth RV, Buerkner P, Hervé M, Love J, Miguez F, Riebl H, Singmann H (2018) Emmeans: estimated marginal means, aka least-squares means. R package version 1.7.0. see cran.r-project.org/package=emmeans, vol. 1, 1 edn

[CR33] Maceljski M, Balarin I (1972). On knowledge of polyphagy and its importance for the silver-Y moth (*Autographa gamma* L.). Acta Entomol Jugoslavica.

[CR34] Menacer K, Cortesero AM, Hervé MR (2021). Challenging the preference–performance hypothesis in an above-belowground insect. Oecologia.

[CR35] Mukerji MK, Harcourt DG (1970). Spatial pattern of the immature stages of *Hylemya brassicae* on cabbage. Can Entomol.

[CR36] Müller C, Agerbirk N, Olsen CE, Boevé J-L, Schaffner U, Brakefield PM (2001). Sequestration of host plant glucosinolates in the defensive hemolymph of the sawfly *Athalia rosae*. J Chem Ecol.

[CR37] Neveu N, Grandgirard J, Nenon JP, Cortesero AM (2002). Systemic release of herbivore-induced plant volatiles by turnips infested by concealed root-feeding larvae *Delia radicum* L.. J Chem Ecol.

[CR38] Oerke E-C (2006). Crop losses to pests. J Agric Sci.

[CR39] Pierre PS, Dugravot S, Ferry A, Soler R, van Dam NM, Cortesero A-M (2011). Aboveground herbivory affects indirect defences of brassicaceous plants against the root feeder *Delia radicum* Linnaeus: laboratory and field evidence. Ecol Entomol.

[CR40] Pierre PS, Dugravot S, Cortesero A-M, Poinsot D, Raaijmakers CE, Hassan HM, van Dam NM (2012). Broccoli and turnip plants display contrasting responses to belowground induction by *Delia radicum* infestation and phytohormone applications. Phytochemistry.

[CR41] Pieterse CMJ, Leon-Reyes A, van der Ent S, van Wees SCM (2009). Networking by small-molecule hormones in plant immunity. Nat Chem Biol.

[CR42] Poelman EH, Broekgaarden C, van Loon JJA, Dicke M (2008). Early season herbivore differentially affects plant defence responses to subsequently colonizing herbivores and their abundance in the field. Mol Ecol.

[CR43] R Core Development Team (2017) R: A language and environment for statistical computing. R Foundation for Statistical Computing, Vienna, Austria.

[CR44] Rasmann S, Turlings TCJ (2007). Simultaneous feeding by aboveground and belowground herbivores attenuates plant-mediated attraction of their respective natural enemies. Ecol Lett.

[CR45] Ratzka A, Vogel H, Kliebenstein DJ, Mitchell-Olds T, Kroymann J (2002). Disarming the mustard oil bomb. Proc Natl Acad Sci USA.

[CR46] Reymond P, Bodenhausen N, Van Poecke RMP, Krishnamurthy V, Dicke M, Farmer EE (2004). A conserved transcript pattern in response to a specialist and a generalist herbivore. Plant Cell.

[CR47] Roessingh P, Städler E, Fenwick GR, Lewis JA, Nielsen JK, Hurter J, Ramp T (1992). Oviposition and tarsal chemoreceptors of the cabbage root fly are stimulated by glucosinolates and host plant extracts. Entomol Exp Appl.

[CR48] Roessingh P, Städler E, Baur R, Hurter J, Ramp T (1997). Tarsal chemoreceptors and oviposition behaviour of the cabbage root fly (*Delia radicum*) sensitive to fractions and new compounds of host-leaf surface extracts. Physiol Entomol.

[CR49] Rojas JC, Wyatt TD, Birch MC (2000). Flight and oviposition behavior toward different host plant species by the cabbage moth, *Mamestra brassicae* (L.)(Lepidoptera: Noctuidae). Journal of Insect Behavior.

[CR50] Rowen E, Kaplan I (2016). Eco-evolutionary factors drive induced plant volatiles: a meta-analysis. New Phytol.

[CR51] Schoonhoven LM, van Loon JJA, Dicke M (2005). Insect–plant biology.

[CR52] Soler R, Bezemer TM, Cortesero AM, van der Putten WH, Vet LEM, Harvey JA (2007). Impact of foliar herbivory on the development of a root-feeding insect and its parasitoid. Oecologia.

[CR53] Stam JM, Kroes A, Li Y, Gols R, van Loon JJA, Poelman EH, Dicke M (2014). Plant interactions with multiple insect herbivores: from community to genes. Annu Rev Plant Biol.

[CR54] Textor S, Gershenzon J (2009). Herbivore induction of the glucosinolate–myrosinase defense system: Major trends, biochemical bases and ecological significance. Phytochem Rev.

[CR55] Vandesompele J, De Preter K, Pattyn F, Poppe B, Van Roy N, De Paepe A, Speleman F (2002). Accurate normalization of real-time quantitative RT-PCR data by geometric averaging of multiple internal control genes. Genome Biol.

[CR56] Zeileis A, Hothorn T (2002). Diagnostic checking in regression relationships. R News.

[CR57] Zohren E (1968). Laboruntersuchungen zu Massenanzucht, Lebensweise, Eiablage und Eiablageverhalten der Kohlfliege, *Chortophila brassicae* Bouché (Diptera, Anthomyiidae). Zeitschrift Für Angewandte Entomologie.

