# Supplementary material for: Leaf-chewing herbivores affect preference and performance of a specialist root herbivore
Source: Oecologia. 2022 Feb 22;199(2):243–55. doi: 10.1007/s00442-022-05132-9 (PMC9226102; doi:10.1007/s00442-022-05132-9)
Supplement: Supplementary file 1 — Supplementary file1 (DOCX 14 KB) [file 442_2022_5132_MOESM1_ESM.docx]

# Supporting information

**Table S1.** Primers used for qPCR of *B. oleracea* leaves and primary roots.

| **Gene acronym** | **Gene amplified** | **Forward primer** | **Reverse primer** |
| --- | --- | --- | --- |
| *AOS* | Bo2g116210 | ACCGCTTGCGACTAGGGATC | CAAAGTCCTTACCGGCGCAC |
| *CYP81F1* | Bo1g004730 | TGTGTCAGAAACGTTCAGGCT | ATGGCACGTCGTATCCTCCG |
| *MYB28* | Bo2g161590 | CGGGAGAGATGAGCACAATACG | CAGCCCTCGAAGTTTCCTATCA |
| *SAR1a* | Bo3g052780 | ATCTCTAGCCACCGTTCCCT | TTCCTGACGATGCTGCACAT |
| *Btub* | Bo2g124350, Bo7g067360, Bo9g059850 | GTCAAGTCCAGCGTCTGTGA | TCACACGCCTGAACATCTCC |
| *Act-2* | Bo5g117040 | ACATTGTGCTCAGTGGTGGA | TCTGCTGGAATGTGCTGAGG |
| *PER4* | Bo7g095750 | TATCCTCTGCAGCCTCCTCA | ACACACAGACTGAAGCGTCC |
| *GADPH* | Bo5g017500 | GCTACGCAGAAGACAGTTGATGG | TGGGCACACGGAAGGACATAC |
| *EF1a* | Bo9g142520 | GGTACCTCCCAGGCTGATTG | TCAGGTAKGAAGACACCTCCTTG |
